# Hybrid treatment of fibroadipose vascular anomaly: A case report

**DOI:** 10.1515/med-2020-0228

**Published:** 2020-09-11

**Authors:** Francesco Stillo, Federica Ruggiero, Antonio De Fiores, Rita Compagna, Bruno Amato

**Affiliations:** Vascular Malformation Unit, Department of Surgery, Istituto, Clinica Guarnieri, Via Tor de’ Schiavi, 139, 00172, Rome (RM), Italy; Department of Clinical and Molecular Medicine, Faculty of Medicine and Psychology, Vascular Surgery Unit, University of Rome La Sapienza, Via di Grottarossa 1035/1039, 00189 Rome, Italy; Department of Public Health, University of Naples “Federico II”, via Sergio Pansini, 5 – 80131, Naples, Italy

**Keywords:** fibroadipose vascular anomaly, ethanol embolization, case report, growth syndromes, PIK3CA mutation

## Abstract

**Background:**

First identified in 2014, fibroadipose vascular anomaly (FAVA) is a very rare type of venous and lymphatic malformation. Marked by tough fibrofatty tissue in the extremities overtaking portions of the muscles, it is associated with constant pain and contracture of the affected extremity. There is a paucity of literature, and no guidelines on treatment procedure are available. This case highlights the role of hybrid treatment with primary ethanol percutaneous ethanol embolization and additional surgery for radicality in excision of FAVA lesions.

**Case summary:**

A 9-year-old girl with FAVA underwent the hybrid treatment. The achievements of complete excision, clinical response, and patient satisfaction in long-term follow-up were assessed. Following the hybrid treatment, the patient experienced significant improvement in pain. Concurrent symptoms of physical limitation, leg swelling, and skin hyperesthesia also improved. The clinical benefit, supported by postoperative physiotherapy, was well stabilized at 6-month follow-up, resulting in complete patient satisfaction at 12- and 36-month follow-ups. No major complications were encountered.

**Conclusion:**

Ethanol embolization plus surgery is a safe, effective, and long-term hybrid treatment of symptomatic FAVA lesions.

## 1 Introduction

New vascular anomalies are continuously identified, considering not only clinical aspects but also radiological and histopathological elements [1,2]. Fibroadipose vascular anomaly (FAVA) was described for the first time in 2014, through a retrospective analysis of 18 patients conducted by Alomari et al. [3]. Nowadays, considering the last published International Society for the Study of Vascular Malformations (known as ISSVA) 2018 classification, this disease is included in the “provisionally unclassified vascular anomalies” [4].

Cases of FAVA are characterized by a unique fibrofatty growing infiltration that is localized within the muscles of the leg (the gastrocnemius muscle is principally afflicted) and atypical phlebectasia, associated with constant pain and contracture of the affected extremity [5,6]. It is mostly encountered in females and is commonly related to somatic mutations in the gene encoding phosphatidylinositol-4,5-biphospate 3-kinase catalytic subunit alpha (PIK3CA) – identified in isolated lymphatic and venous tissue of affected patients, similar to that in other complex overgrowth vascular syndromes. In young girls, its growth activation is probably related to hormonal factors [7,8].

Vascular surgery consultation is often required for patients with FAVA; therefore, vascular surgeons should be aware of this new entity to distinguish it from the classical venous malformations or tumors of the lower extremities and to avoid delay in diagnosis and/or initiation of inappropriate treatments [9].

## 2 Case presentation

### 2.1 History of present illness

In June 2016, a 9-year-old girl (elementary school student of Caucasian race, weighing 42 kg) was referred for consultation with an interdisciplinary team in the Vascular Anomalies Center of Guarneri Clinic (Rome, Italy) to address a painful and progressively increasing mass in the right thigh. The consultation team included vascular surgeons, interventional radiologists, hematologists, oncologists, plastic surgeons, and pathologists.

### 2.2 History of past illness

The patient had an unremarkable medical history.

### 2.3 Physical examination

**Figure 1:**
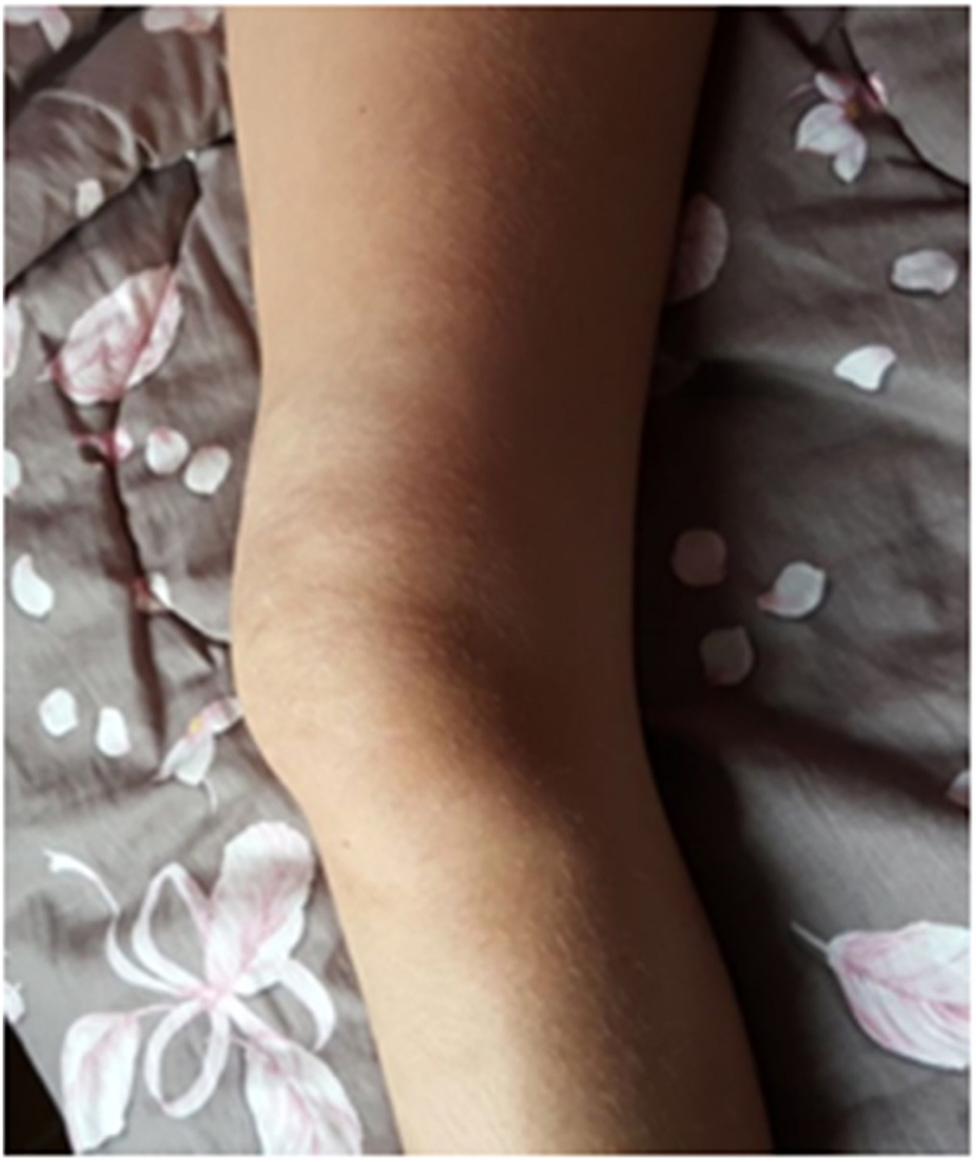
Injury of the vastus medialis muscle, which was visible on the clinical examination of the lower thigh. Parental consent was obtained for publication of this clinical photograph.

At clinical examination, the girl showed functional impairment of the right knee (limited flexion of 30%), muscle contracture, and localized pain (Figure 1). In the days before her hospitalization, the reported daily mean visual analog scale (VAS) score for chronic pain was 7  ±  1. Neurologic examination revealed normal patellar tendon reflex, full strength of the surrounding musculature, and normal superficial and deep sensitivities. Dermatological examination revealed normal skin characteristics.

It was reported that no therapeutic procedure had been carried out previously, and the pain symptoms had been controlled with pain relievers, when necessary.

### 2.4 Laboratory examinations

Laboratory blood tests for coagulation were performed, according to the high rate of variations in coagulation states that are found in patients with vascular anomalies. No abnormalities were observed in our young patient.

### 2.5 Imaging examinations

**Figure 2:**
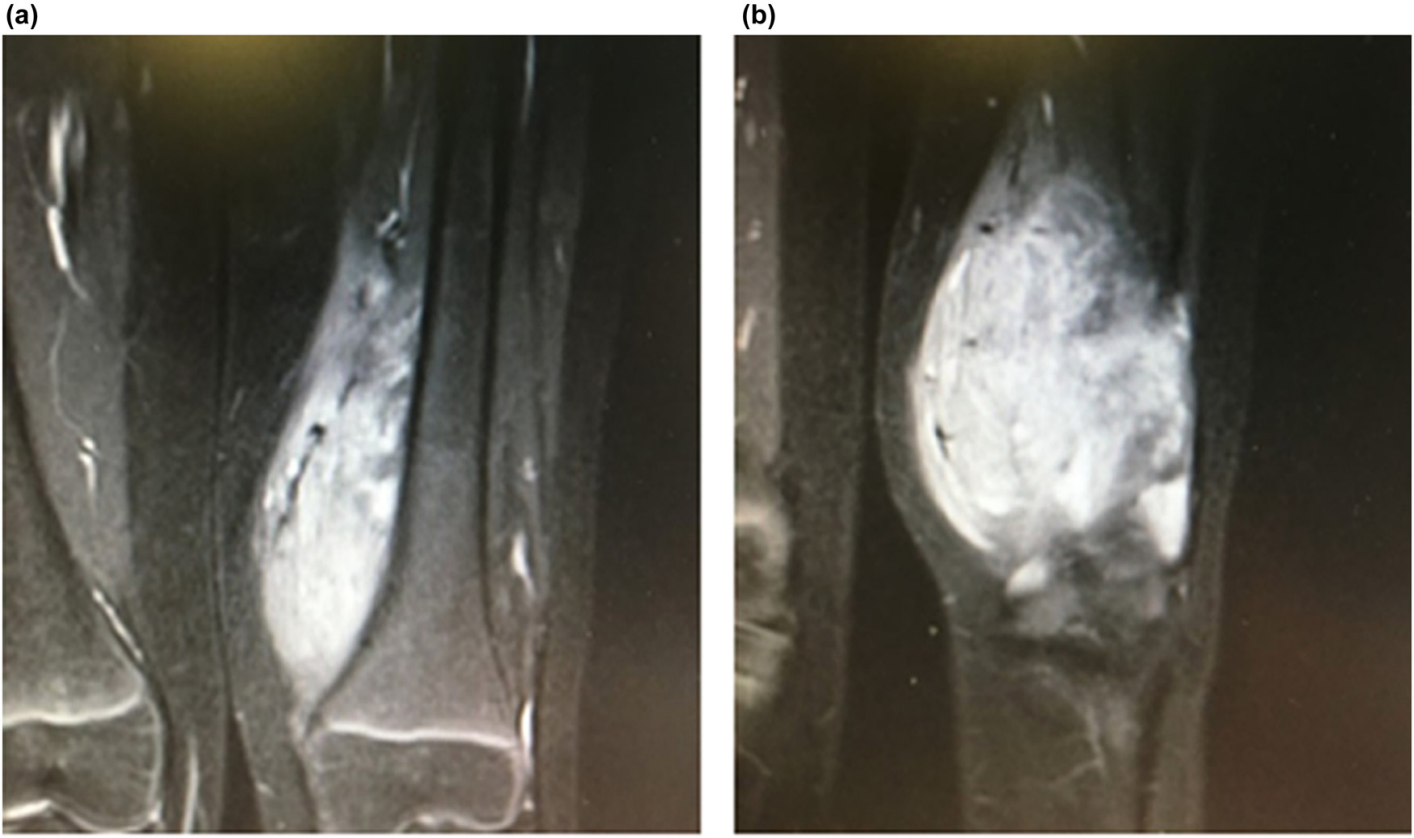
Preoperative magnetic resonance imaging. (a) Focal mass-like FAVA, which produced progressive thigh pain over the last year; (b) the sagittal image shows maximal mass hyperintensity in T2-weighted images.

In addition to clinical signs, diagnosis of FAVA was based on both ultrasound and magnetic resonance imaging (MRI) examinations. Both imaging modalities showed a complex mass of 11 cm × 8 cm × 8 cm in the right thigh, a finding compatible with FAVA malformation, located in the vastus medialis muscle and marginally in the rectus femoral muscle. The T1-weighted MRIs showed heterogeneous and hyperintense signal related to the fat component (Figure 2), and the injection of intravenous contrast clearly revealed the malformed venous component. The solid mass was found to have partially replaced the normal vastus medialis muscle fibers, with fibrofatty overgrowth and varied appearances of clusters of thick-walled muscular vessels; multiple soft-tissue planes were found to be involved as well, showing hypoechoic intralesional clots or development of phleboliths, with low-flow venous parameters that were also barely detectable by Doppler sonography.

Following discussion and consensus by the interdisciplinary team, differential diagnoses of other commonly confused vascular lesions (i.e., venous or arteriovenous malformations or vascular tumors) were ruled out.

### 2.6 Strategy development

The primary end point of this patient’s treatment was radical FAVA treatment. The secondary end points were pain relief (assessed by VAS) and recovery of functional impairments [10,11].

Previous clinical experiences in the literature describe the frequent use of sclerotherapy and percutaneous cryoablation procedures for FAVA [12,13,14] and the high risk of blood loss and nervous system damage related to primary surgery in FAVA [6]. Thus, the multidisciplinary team proposed an original hybrid treatment strategy of primary percutaneous ethanol embolization of the FAVA and a secondary surgical excision of the mass.

**Informed consent statement:** Written consent was obtained from both parents of the patient, because she was a minor at the time of hospitalization.

**Institutional review board statement:** The Institutional Review Board of Guarnieri Clinic provided approval for this study (IRB No. 19/1721).

## 3 Final diagnosis

The final consensus diagnosis made by the multidisciplinary team was FAVA.

## 4 Treatment

Preoperatively, the available MRI results were reviewed, to correlate the symptomatic sites. Doppler ultrasound (Logiq E9; GE Healthcare, Chicago, IL, USA) was performed to map (with skin marker) the patient’s sites of pain to identify the areas of most intense vascularization and facilitate the procedure of percutaneous embolization [15].

Intraoperatively, under spinal cord anesthesia and following sterile preparation, we performed ultrasound-guided percutaneous placement of a single 19-G angiocatheter needle in the central position along the long axis of the FAVA. First, iodinated contrast medium (Conray®; Liebel-Flarsheim Company LLC, Raleigh, NC, USA) was injected to fluoroscopically evaluate the extent of the FAVA; second, 4 mL of absolute ethanol was injected into the FAVA lesion. All procedures were performed under epidural anesthesia and a nerve-block catheter was applied for 24 h to control postprocedural pain and discomfort.

Postoperatively, per our usual ethanol scleroembolization protocol, 1 week of prednisone was administered at a dose of 2 mg/kg/day and a 2% cortisone cream was applied twice a day to the skin of the treated area for the same period.

At 1-month sonographic follow-up, the outcome of percutaneous embolization was assessed by ultrasound Doppler; an absence of visible patent vessel in the lesion and postembolization FAVA mass reduction (from the original size to 9 cm × 7 cm × 6 cm) were observed. In this time period, functional impairment changed as well, with the original limits in foot support and knee flexion showing partial improvements (from 30% to 20%). Within 8 week after the embolization, the daily mean pain and discomfort improved (from the original VAS score of 7 to 3).

**Figure 3:**
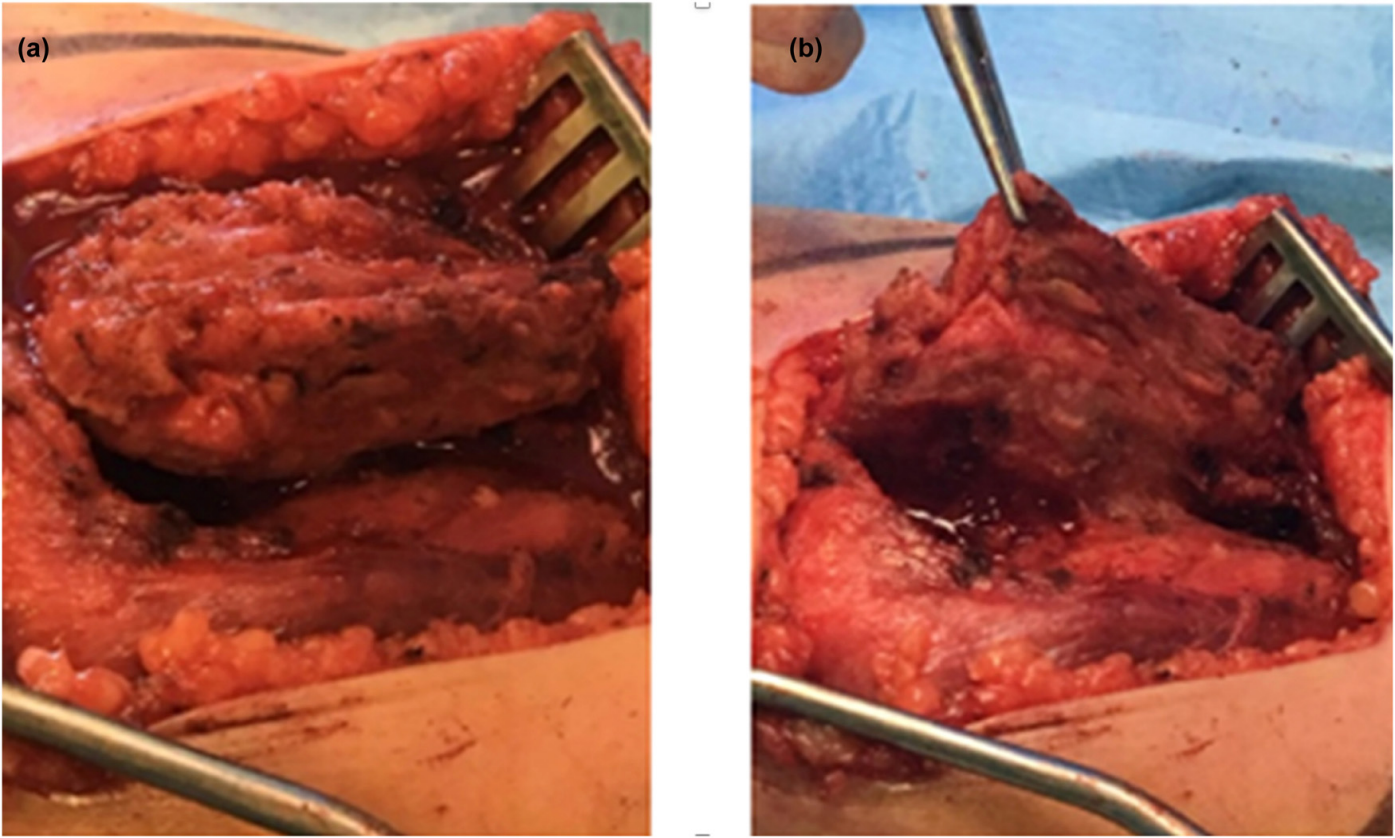
Intraoperative images from the resection surgery. (a) Isolation of the mass; (b) mass excision.

At 2 months after the ethanol embolization, the patient underwent the second treatment – surgery for resection of the residual FAVA and of the partial involved muscle. Under spinal cord anesthesia, a marginal resection of the mass was performed through a medial thigh incision (Figure 3a). The mass had two components, namely, a fibrotic residual venous malformation (embolization related) within the vastus medialis muscle and surrounded by normal muscle tissue, and a fatty component (Figure 3b).

The excised affected tissues were collected for subsequent pathological examinations, including histological and genetic. For the latter, fresh samples were frozen and then processed for DNA extraction (QIAamp DNA Mini Tissue Kit; Qiagen, Germantown, MD, USA) and subjected to the next-generation DNA sequencing.

At the end of the resection procedure, an elastic containment dressing was applied to the affected limb and kept in place for 1 week. The patient was discharged with prescription for a 7-day course of nonsteroidal anti-inflammatory medication (Paracetamol). At 2-week postsurgery, the patient attended follow-up, showing no new symptoms and receiving recommendation for physiotherapy evaluation.

## 5 Outcome and follow-up

**Figure 4:**
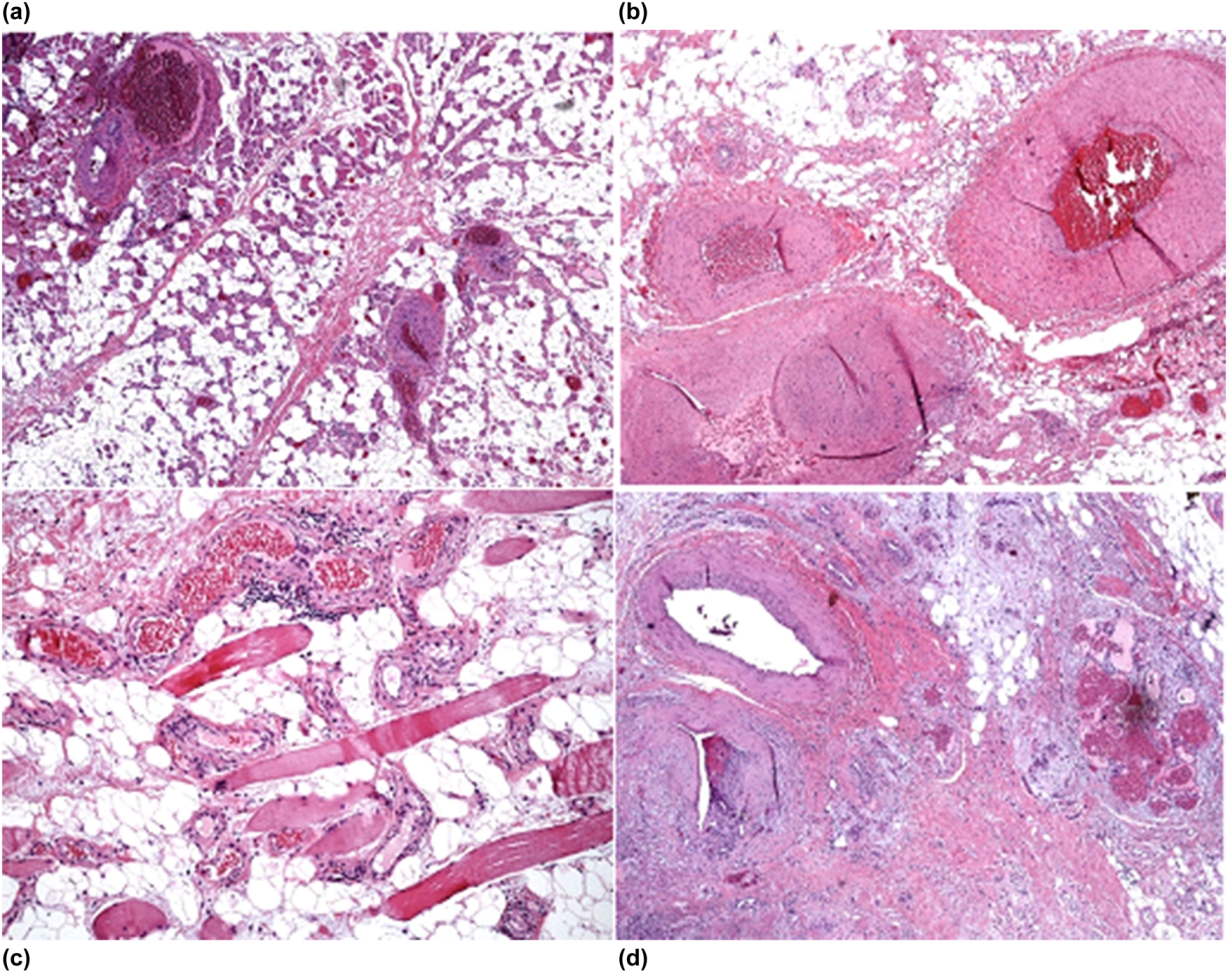
Histological analyses of the resected tissues. (a) Fibrous tissue; (b) clusters of thin-walled venous channels; (c) adipose tissue; (d) lymphoid aggregates within the skeletal muscle. Magnification: 40×; stain: hematoxylin and eosin.

The histological examination (Figure 4) showed the lesion to be composed mostly of predominantly dense adipose tissue, fibrous tissue, and lymphoplasmacytic aggregates within atrophied skeletal muscle with a cluster of fibrotic venous channels, sometimes excessively muscularized and with an anomalous lymphatic vascular component. In addition, organizing thrombi, lymphatic foci and nerves encircled by dense fibrous tissue were observed, and the latter being grouped among atrophied skeletal muscle with entrapped nerves.

The genetic examination showed three mutations in the PIK3CA gene, namely, *p.E542K*, *p.E545K*, and *p.H1047L*. These findings are similar to previous observations reported in the literature, which allowed for FAVA to be classified among the PIK3CA-related overgrowth syndromes [7]. The main procedural complications that have been described after embolization, such as local pain, skin necrosis, peripheral or pulmonary embolism, and coagulopathy, did not develop in our case nor did any complications that have been observed during and after surgical procedures for FAVA, such as local pain, blood loss, nerve injury, and surgical site infections.

We collected the patient’s responses to an inventory of pain, clinical response, and satisfaction, prior to each procedure and at each follow-up visit. The patient attended in-clinic follow-up visits at 1, 2, 6, 12, and 36 months after the hybrid procedure. Doppler ultrasound was performed at each visit. Also, every follow-up visit included documentation of concurrent symptoms, like local swelling, pain, functional restrictions (i.e., mobility, range of motion, weight-bearing exercise, and gait anomaly), and skin hyperesthesia. The postprocedure local swelling, pain, and discomfort resolved within 8 weeks after embolization and within 4 weeks after the resection surgery. The patient had no infection or skin breakdown. She described skin hyperesthesia and mild numbness, present first on the embolization area and successively on the area of surgical excision and along the nerve distribution of the leg; all improved postprocedurally (in 8 weeks after the ethanol embolization and after 3 months for the resection) and were considered minor complications. The limb mobility, weight-bearing exercise, and improvement in gait anomaly progressed gradually accompanied by physiotherapy; full extension of the knee and normal gait were reached after 6 months from the time of the hybrid procedure, remaining unchanged and stable at following clinical checkups.

**Figure 5:**
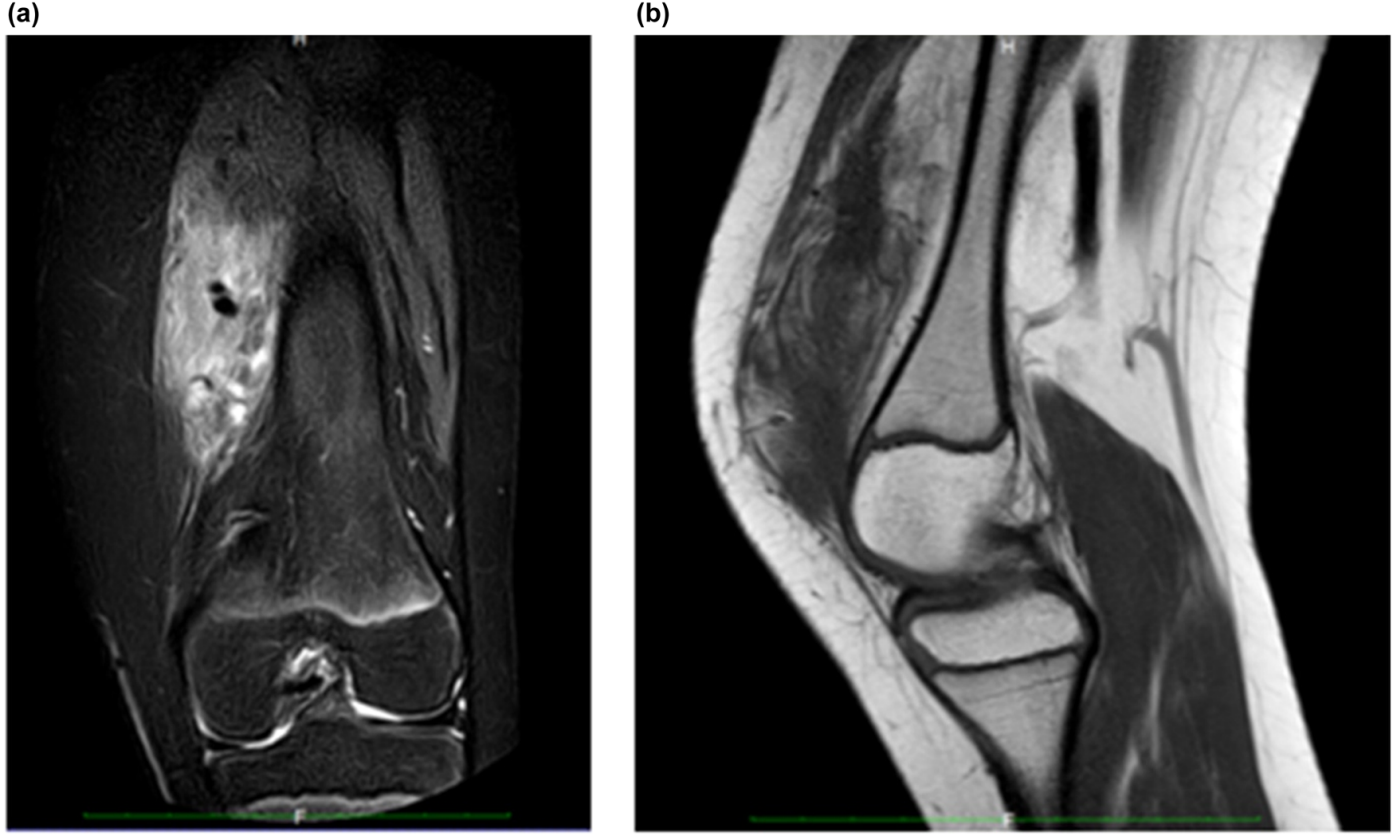
Pre- and postoperative magnetic resonance imaging showing significant improvement in the adipose and vascular components. (a) Preoperative; (b) at 12 months’ postoperative.

**Figure 6:**
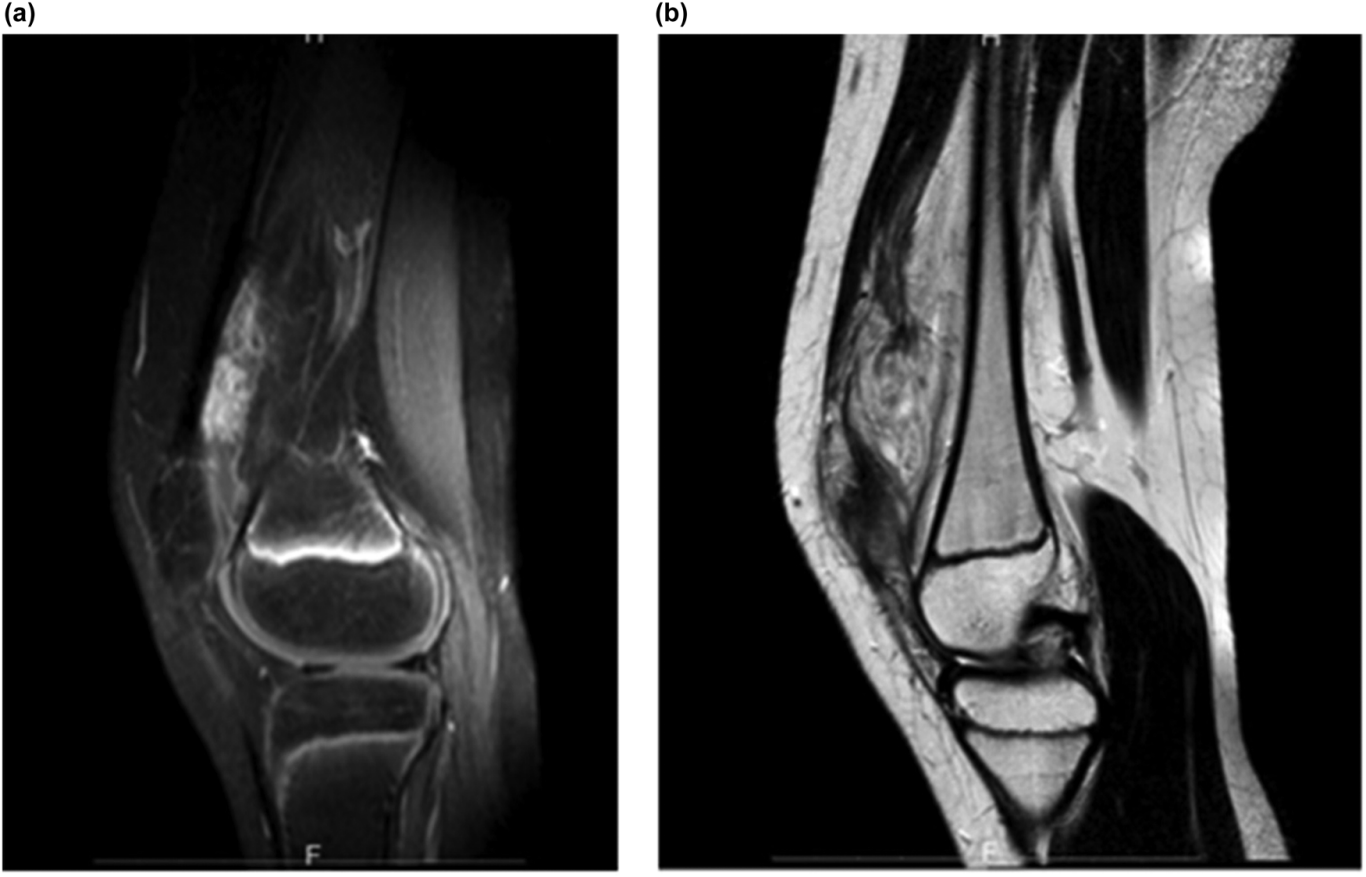
Follow-up magnetic resonance imaging at 3 years postoperative. (a and b) Both images show the minimal fibroadipose gaps in the vastus medialis muscle.

Patient satisfaction with the hybrid procedure was also assessed after the embolization, after the resection, and at 6-, 12-, and 36-month follow-ups; each time, the patient noted the grade of “very satisfying.” To verify the possible recurrence of the FAVA, the affected limb was also checked with ultrasound Doppler and MRI at 12 months (Figure 5) and 3 years (Figure 6) out from the treatment; both follow-ups found some minimal and stable fibroadipose gaps in the vastus medialis muscle, at every control, which testified to the long-term absence of recurrent FAVA, even during the stage of pubertal development in our young patient.

## 6 Discussion

Although FAVA was only recently identified as a pathology, the clinical, genetic, and histological aspects and the imaging characteristics of this rare malformation remain to be fully defined [3]. Common symptoms of this pathology include severe pain and difficulty moving the affected limb, moderate enlargement of the limb with visible veins, and muscle contracture; the disease is most often diagnosed in older children and teenagers [5].

In descending order, FAVA cases involve the calf, forearm/wrist, and thigh. The most common symptom is severe pain. Lesions located in the posterior compartment are commonly associated with restricted ankle dorsiflexion [6]. In comparison with other vascular malformations, which manifest episodic pain typically and involve dermis, FAVA is characterized by a constant and often intense pain and lack of dermal involvement [6].

Genetic studies are increasingly used to complete the diagnosis of FAVA. Alomari et al. [3] reported on eight patients affected by FAVA who presented with somatic mutations of PIK3CA but only in the malformed lymphatic component of the resected tissue samples. From a histological point of view, FAVA is difficult to distinguish from the phosphatase and tensin-homolog (*PTEN* gene) soft-tissue hamartoma. This pathology is revealed by the presence of a syndromic picture with arteriovenous communications and mutation of the *PTEN* gene [7].

Finally, the histological examination can confirm FAVA by the presence of a dominant adipose component infiltrated into the muscle and associated with fibrosis, with frequent accompaniment by phleboliths in the venous vessels. This anomaly causes atrophy of the muscle fibers themselves and extends around the nerve trunks. In the soft tissues surrounding the muscle, the lymphoplasma cell infiltrations and foci of bone metaplasia may also occur. Otherwise, skin alterations are usually unremarkable [5].

For FAVA, the imaging investigations show both newly formed fibroadipose tissue in the muscular framework and a malformative component, mainly of a venous type. Ultrasonographic gray scale and color Doppler studies reveal an abnormally dilated and increased number of vessels in the venous district, no increase in flow in the arterial one, and finally the possible presence of phleboliths (which cause part of the pain via the venous dilatations). MRI has been the most widely used imaging modality for FAVA; the images typically highlight the epicenter of the lesion in the intramuscular area. Furthermore, MRI investigation reveals the malformation extension into the surrounding soft tissues, with the T1-weighted images indicating the presence of an adipose component and the contrast agent showing a venous component [3].

Regarding the treatment of FAVA, its structural component (venous–lymphatic anomalies, in addition to the adipose and fibrous components) inherently makes sclerotherapy useful; but such is only effective in the treatment of the vascular malformations. As reported in the recent literature, sclerotherapy provides only temporary relief, acting on the venous component, but the means for a global and permanent treatment have not yet been fully determined [3,5]. To eliminate the fibrofatty component that includes and pinches the nerves, a more radical result is obtained by surgical removal of the mass, including the adipose and vascular components and sometimes the corresponding muscle, with extensive and debilitating tissue resections. Surgical treatments are then accompanied by medication, to temporarily limit pain, and by physiotherapy [6].

In the last years, cryoablation has been suggested as a new treatment of FAVA [13,14]. The advantage of this therapy would be related to the shorter hospitalization time compared to traditional surgery and a greater involution of the ablation area. However, the limited number of patients treated with it to date, the large number among those patients who had previously undergone sclerosing or surgical therapy, and the presence of several clinical recurrences do not yet allow for consideration of this approach as an elective therapy for FAVA [14,16]. Recently, experiences with rapamycin (Sirolimus), an immunosuppressant agent used to prevent organ transplant rejection, have been described as effective in improving the quality of life in some people with FAVA [17].

The single procedures proposed so far in the treatment of FAVA have shown some limits linked to their poor or incomplete radicality (e.g., sclerotherapy, embolization with ethanol, and cryoablation) or their relation to major complications (e.g., surgery and rapamycin). Our proposal of a sequential hybrid treatment with ethanol embolization and resection surgery has instead allowed for a result of long-term radicality and the absence of major complications in the treatment of such a complex vascular anomaly as FAVA.

## 7 Conclusion

FAVA is a rare complex vascular anomaly, with distinctive clinical and imaging features. Despite the low number of patients studied and reported in the international literature, surgical therapy seems to be the best therapeutic choice because it reduces pain and avoids further recurrences. Unfortunately, primary surgery for FAVA carries the risk of major complications, such as blood loss and nerve injuries. We believe, therefore, that hybrid treatment with primary ethanol percutaneous embolization and secondary resection surgery offers advantages in terms of both malformation and fatty mass ablation, without surgery-related complications.

Our FAVA case who underwent the hybrid treatment experienced a clinical response that was very satisfactory and without long-term recurrence or any major procedure-related complications. This case suggests that the hybrid treatment may be considered as an effective and safe option for radical resolution of symptomatic FAVA. However, additional clinical experiences and a larger number of patients are needed to provide a sufficiently comprehensive evaluation of this procedure.
